# COVID-19 Variant Surveillance and Social Determinants in Central Massachusetts: Development Study

**DOI:** 10.2196/37858

**Published:** 2022-06-13

**Authors:** Qiming Shi, Carly Herbert, Doyle V Ward, Karl Simin, Beth A McCormick, Richard T Ellison III, Adrian H Zai

**Affiliations:** 1 Center for Clinical and Translational Science UMass Chan Medical School Worcester, MA United States; 2 Department of Population and Quantitative Health Sciences UMass Chan Medical School Worcester, MA United States; 3 Department of Medicine UMass Chan Medical School Worcester, MA United States; 4 Department of Microbiology and Physiological Systems UMass Chan Medical School Worcester, MA United States; 5 Center for Microbiome Research UMass Chan Medical School Worcester, MA United States; 6 Molecular, Cell, and Cancer Biology UMass Chan Medical School Worcester, MA United States

**Keywords:** geographic information science, GIS, COVID-19, SARS-CoV-2, variants, surveillance, dashboard, web mapping, public health, web-based information, digital health, epidemiology

## Abstract

**Background:**

Public health scientists have used spatial tools such as web-based Geographical Information System (GIS) applications to monitor and forecast the progression of the COVID-19 pandemic and track the impact of their interventions. The ability to track SARS-CoV-2 variants and incorporate the social determinants of health with street-level granularity can facilitate the identification of local outbreaks, highlight variant-specific geospatial epidemiology, and inform effective interventions. We developed a novel dashboard, the University of Massachusetts’ Graphical user interface for Geographic Information (MAGGI) variant tracking system that combines GIS, health-associated sociodemographic data, and viral genomic data to visualize the spatiotemporal incidence of SARS-CoV-2 variants with street-level resolution while safeguarding protected health information. The specificity and richness of the dashboard enhance the local understanding of variant introductions and transmissions so that appropriate public health strategies can be devised and evaluated.

**Objective:**

We developed a web-based dashboard that simultaneously visualizes the geographic distribution of SARS-CoV-2 variants in Central Massachusetts, the social determinants of health, and vaccination data to support public health efforts to locally mitigate the impact of the COVID-19 pandemic.

**Methods:**

MAGGI uses a server-client model–based system, enabling users to access data and visualizations via an encrypted web browser, thus securing patient health information. We integrated data from electronic medical records, SARS-CoV-2 genomic analysis, and public health resources. We developed the following functionalities into MAGGI: spatial and temporal selection capability by zip codes of interest, the detection of variant clusters, and a tool to display variant distribution by the social determinants of health. MAGGI was built on the Environmental Systems Research Institute ecosystem and is readily adaptable to monitor other infectious diseases and their variants in real-time.

**Results:**

We created a geo-referenced database and added sociodemographic and viral genomic data to the ArcGIS dashboard that interactively displays Central Massachusetts’ spatiotemporal variants distribution. Genomic epidemiologists and public health officials use MAGGI to show the occurrence of SARS-CoV-2 genomic variants at high geographic resolution and refine the display by selecting a combination of data features such as variant subtype, subject zip codes, or date of COVID-19–positive sample collection. Furthermore, they use it to scale time and space to visualize association patterns between socioeconomics, social vulnerability based on the Centers for Disease Control and Prevention’s social vulnerability index, and vaccination rates. We launched the system at the University of Massachusetts Chan Medical School to support internal research projects starting in March 2021.

**Conclusions:**

We developed a COVID-19 variant surveillance dashboard to advance our geospatial technologies to study SARS-CoV-2 variants transmission dynamics. This real-time, GIS-based tool exemplifies how spatial informatics can support public health officials, genomics epidemiologists, infectious disease specialists, and other researchers to track and study the spread patterns of SARS-CoV-2 variants in our communities.

## Introduction

COVID-19 has infected 219 million people resulting in over 4.5 million deaths as of October 2021 [1]. The pandemic has highlighted the role of geographic mapping technologies to provide easy-to-understand indicators such as new cases, confirmed tests, and inpatient rates using spatiotemporal visualizations [2]. For example, public agencies [3] and news media [4,5] have used these visualizations to inform the public about the spread of COVID-19 and explain why officials recommend adopting intervention measures such as mask mandate and vaccinations [6].

Health science has long leveraged Geographical Information System (GIS) spatial analysis and applications [7]. GIS offers an interactive and efficient approach to revealing meaningful patterns and associations [8] that would be otherwise difficult to visualize using traditional figures and tables. As a result, researchers are increasingly using spatial analysis to study the impacts of COVID-19 [9,10] or understand the relationship between COVID-19 cases and sociodemographic or economic data and vaccination rates in local environments [11-13].

With the constant technological evolution of GIS platforms, many geospatial online dashboards are available for monitoring COVID-19 worldwide [14,15]. The COVID-19 dashboard developed by John Hopkins University is arguably the most popular and stands out for its effectiveness at capturing new cases around the globe [16]. Meanwhile, the Centers for Disease Control and Prevention’s National Healthcare Safety Network has also developed a geospatial dashboard for COVID-19 infection data analysis and prevention [17]. Most GIS systems, however, track data with coarse geographic resolution (eg, state or county). Further, few GIS systems track the spatiotemporal transition dynamics of SARS-CoV-2 variants [18].

The impact of using genomic epidemiology to monitor the COVID-19 pandemic has been profound, as it provides unprecedented detail into the appearance and global dissemination of SARS-CoV-2 variants [19-24]. In addition, phylogenetic-epidemiological analysis has enabled the reconstruction of super-spreader events [25]. Notably, these analyses have illustrated the power of combining epidemiological analytics with deep viral genome sequencing to gain fundamental insights into mutational dynamics and transmission properties [26]. Meanwhile, researchers have developed visualization tools to analyze phylogenetics spatially [27-29], albeit with a coarse geospatial resolution due to poor detail in data collection.

A detailed analysis of the demographic and social determinants of disease risk provides insights into how social characteristics impact health risks and disparities [30-32]. For many infectious diseases, populations of lower socioeconomic status tend to be associated with higher disease prevalence [33-35]. Typically, GIS dashboards fail to incorporate the socioeconomic correlates of health that could provide a deeper understanding of transmission dynamics when combined with geographic and genomic data.

This paper presents the development of the University of Massachusetts’ Graphical user interface for Geographic Information (MAGGI) variant tracking system, a GIS dashboard that integrates genomics and socioeconomic data into a high-resolution geographical dashboard. Our aims for developing MAGGI were to (1) track the transition dynamics of SARS-CoV-2 variants across space and time, (2) identify geographical areas at high risk for transmission using variant cluster risk analysis, and (3) assess socioeconomic risk factors within these high-risk areas.

## Methods

### Ethics Approval

This study was approved by the University of Massachusetts (UMass) Chan Medical School Institution Review Board (protocol H00021561).

### Data Source

Data incorporated into MAGGI originates from multiple sources, including the American Community Survey [36], Massachusetts Department of Public Health (DPH) [37], Centers for Disease Control and Prevention [38], and the UMass Chan Medical School clinical research data warehouse (RDW). The RDW combines data from the UMass Memorial electronic health record system from Epic, Allscripts, and Soarian. Next, we used the 2018 American Community Survey 5-year data to extract zip code–level social demographic data, including race, ethnicity, median family income, housing, language, poverty, and population density. We downloaded the ZIP Code Tabulation Areas (ZCTA) data from the census, and ZCTA are what we used in the MAGGI system. However, we used zip code as a proxy of ZCTA since the latter will be the same as its zip code in most cases and the term zip code is more commonly used and known. Finally, we obtained the COVID-19 vaccination rate data from the Massachusetts DPH and the social vulnerability index from the Centers for Disease Control and Prevention [33].

Remnant positive SARS-CoV-2 test patient samples (swab and saliva) from the UMass Memorial Health Care system were collected and archived by the UMCCTS Biospecimen bank. Sample aliquots were transferred to the UMass Center for Microbiome research for SARS-CoV-2 genomic extraction and sequencing. Sequencing libraries were prepared using NEBNext ARTIC SARS-CoV-2 FS Library Prep Kits and sequenced on the Illumina NextSeq 500 platform as 75nt paired-end reads per manufacturer protocols. Sequence data were analyzed using the Cecret workflow developed at the Utah Public Health Laboratory that provides SARS-CoV-2 genomic sequence and lineage determinations [39]. The genomic results were then linked to patient data.

### Geocoding

Geocoding is the technology used to transform physical street addresses into geographic coordinates such as latitude and longitude, enabling us to place markers on the map. After determining the SARS-CoV-2 genomic sequence and lineage, we geocoded the addresses extracted from electronic medical records using the ArcGIS Pro Geocode function on the secure UMass Amazon Web Service private cloud. All geocoding processes used a local geolocator from the Environmental Systems Research Institute (ESRI). Approximately 10%-15% of the extracted addresses contained errors or represented recent addresses that the ESRI geolocator had yet to capture; we manually corrected these errors with GPS coordinates obtained from Google Maps search results. Next, we randomly geocoded Post Office Box addresses to the area defined by the corresponding zip code. Last, we deidentified the geocoded layer to include only longitude and latitude, strain lineage, sample collection date, and variant type to further protect patient information.

### Data Integration

**Figure 1:**
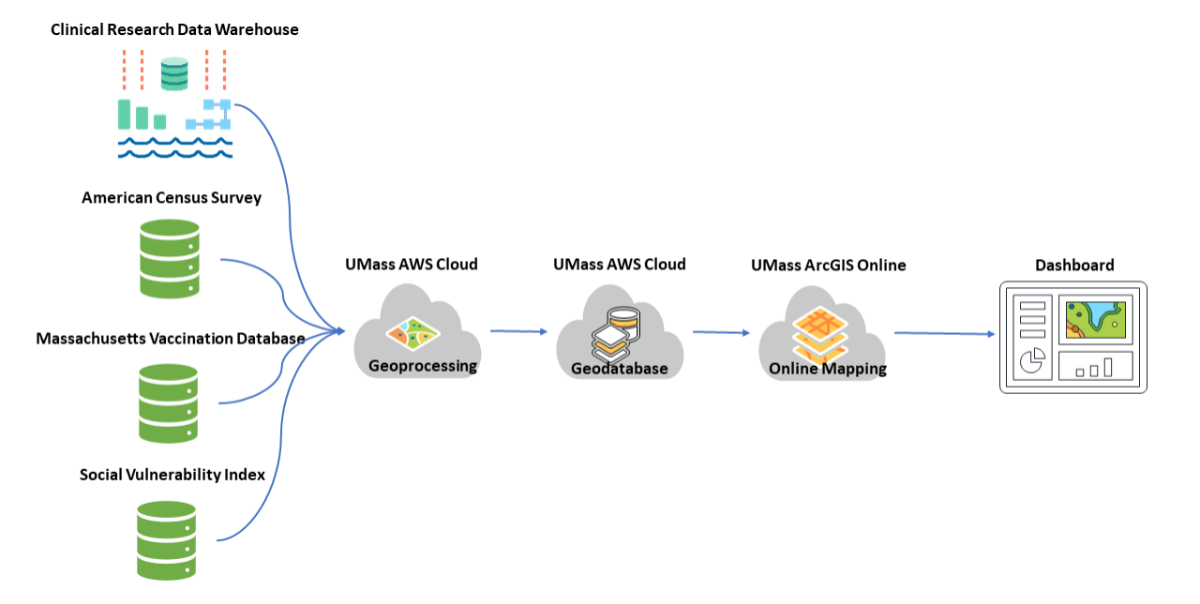
Dashboard workflow and technology stack. UMass: University of Massachusetts; AWS: Amazon Web Service.

The UMass Memorial Health Care System (UMMHC) is the primary health care provider in Central Massachusetts and has used the Epic electronic health record system [40] beginning in 2017. The Epic Clarity database is our primary source for clinical and demographic data. Viral genomic data resides in a separate database. Consequently, we created a Structured Query Language Server database to link genomic data with clinical data using a universal identifier number. This Structured Query Language database remits data to ArcGIS Pro for geocoding. The polygon geometry from zip code and census tracts was integrated into the geodatabase project. Finally, we designed the geodatabase to organize and store spatial databases, tables, and vector data sets. The geodatabase combines geocoding results, social demographics, social vulnerability, and vaccination data, and then passes the data to ArcGIS Online for web-based mapping (Figure 1).

### Data Visualization and Dashboard Development

**Figure 2:**
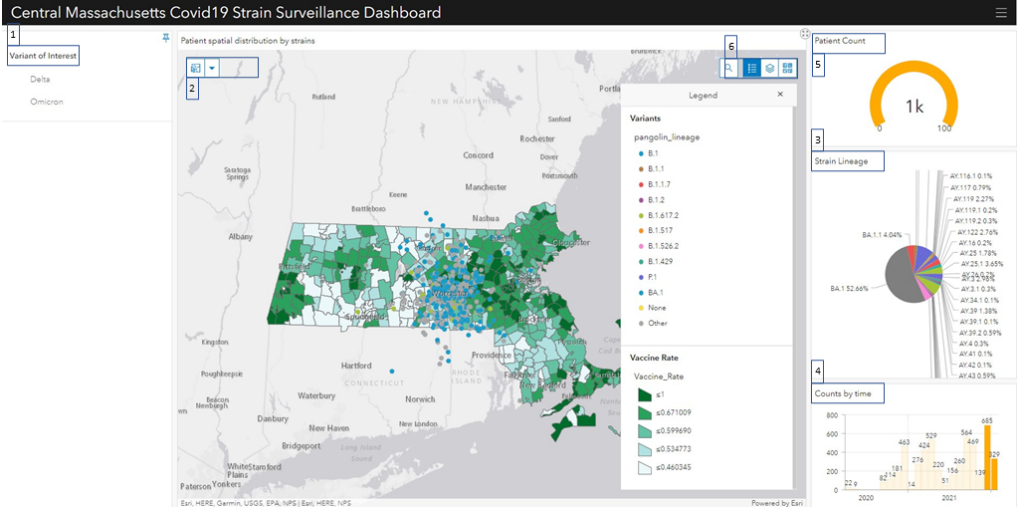
The Massachusetts’ Graphical user interface for Geographic Information (MAGGI) application user interface.

ArcGIS Online, the ESRI web-based mapping software, enables users to build interactive web maps with user interface instead of JavaScript programming [41]. We created the web map to visualize spatial layers with this tool. The layers include the geocoded results of variant type, socioeconomic data, vaccination status, and social vulnerability index data. ArcGIS Dashboard, the essential component to conveying information by presenting location-based analytics using intuitive and interactive data visualization, was used to add interactive charts and functionalities based on the web map. We leveraged the dashboard's versatility to enable users to render a variety of charts and selections, which included the following features (item numbers below correspond to the boxed numbers in Figure 2).

- (1) Variants Selection Panel: We built this variant selection panel using the Category Selector function provided by the ArcGIS dashboard. This panel enables researchers to quickly filter the data to the genomic variants of interest (eg, Delta and Omicron).
- (2) Spatial Selection Tool: We leveraged the Spatial Selection method in the layer actions section provided by the ArcGIS dashboard to enable the interconnection between the genomic variants and socioeconomic layers. This function allows users to investigate patients in selected geographical units such as zip codes and census units. This tool is useful when genomic epidemiologists study and compare variant population trends through various geolocations.
- (3) Strain Lineage Pie Chart function: We created this pie chart using the ArcGIS Pie Chart function to determine the lineage distribution proportion at a given time and zip code. It helps measure the dominated variants and calculate relative risk (RR) in a specific time and space.
- (4) Time Serial Chart: We built this chart using the ArcGIS Serial Chart function to render sequenced data by time series. It displays the count of patients by month and works with other selection criteria simultaneously to study the variant transmission dynamic across space and time.
- (5) Patient Count: Using the ArcGIS Gauge function, we added the patient count gauge to track patient count based on current selection criteria.
- (6) Layer Toggle: We enabled the Basemap Switcher function in the ArcGIS dashboard. This function allows users to change the background map among the socioeconomic layers, social vulnerability index, and vaccination layer. In addition, this function helps researchers easily switch and draw potential correlations between layers.
- Cluster Detection: We used the Getis-Ord Gi* hot spot analysis [42] provided by ArcGIS Online to detect the spatial cluster of the variant.

### Data Security and Sharing

Users must log in to the ArcGIS Online application via a single sign-on with Microsoft multi-factor authentication technology [43]. Access to the dashboard is based on membership or association with the studies, ensuring that only authorized study team members will see the specific study-related dashboard upon log-in. ArcGIS Online offers several grouping categories to restrict access to appropriate users only: organization, groups within an organization, collaborators with an organization, or everyone (public) [44]. For MAGGI, we used this security protocol to set up private groups for local health department administrators to access only data from their respective jurisdictions. Thus, for example, members of the Massachusetts Fitchburg DPH group can only visualize data from the 11 towns managed by their department. In contrast, administrators from the Massachusetts DPH can access and visualize data from the entire state of Massachusetts.

## Results

### User Interface

An interactive dashboard provides the user interface that allows researchers to study the spatiotemporal trend of the variants as they work through the investigation process. The user interface depicting data relating to SARS-CoV-2 variants from patient infections between May and October 2001 is presented (Figure 2).

### Examples of Use Cases

#### Use Case 1: Variant Surveillance

MAGGI enables members of public health agencies in Massachusetts and genomic epidemiologists to monitor regional viral spread patterns and the emergence of potential new variants and study the impact of new interventions, including the effectiveness of new policy implementations. For example, genetic epidemiologists use MAGGI to study the relationship between genetic drifts in the genome of SARS-CoV-2 and transmission rate at the local level, thereby revealing unsuspected clusters and evidence for or against suspected transmissions.

#### Use Case 2: Spatiotemporal Cluster Detection

**Figure 3:**
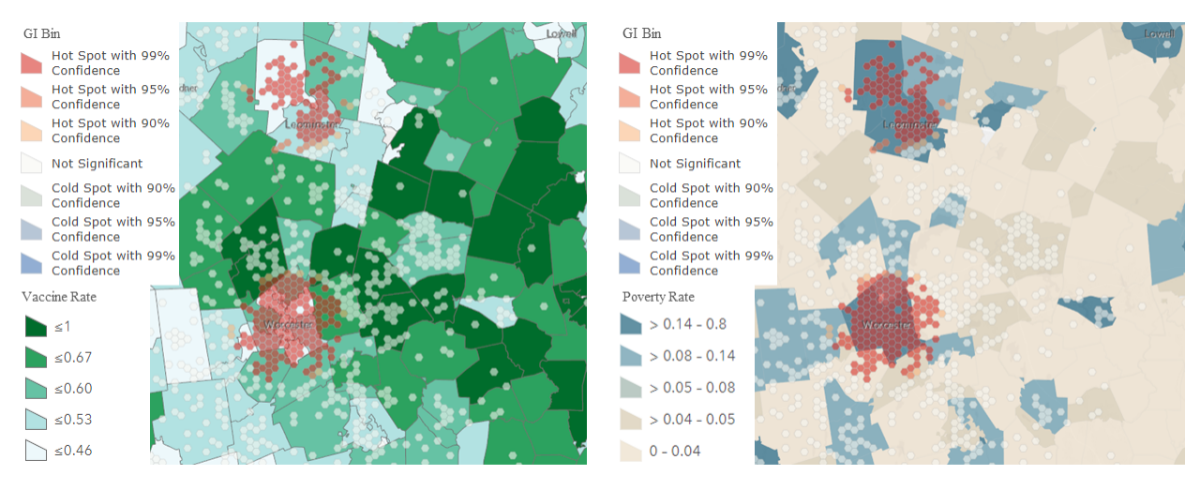
Hot spot analysis (Getis-Ord Gi*) layer overlapping with the social and vaccination determinants layer.

The Hot Spot Analysis tool calculates the cluster risk using the Getis-Ord Gi* statistic [42]. This calculation’s resulting z scores and *P* values inform us where to locate areas with high- or low-value clusters spatially. This tool functions by comparing the z scores and *P* values between any location with its neighbors. Regions labeled as “hot spots” must have a statistically significant score compared to their surrounding neighbors. Based on the cluster risk analysis (Getis-Ord Gi*) results, our first use case objective was to explore the social and vaccination determinants associated with detected clusters. Clusters were identified in the Worcester and Leominster areas served by UMMHC and co-located with areas of lower socioeconomic status and low vaccination rates (Figure 3).

Users can further investigate the dissemination of emerging variants. First, a user specifies the variant of interest and selects the zip codes from the map. The map then updates to visualize the infections meeting the criteria. The user then visualizes the distribution in the time series chart and individually inspects each interval by making the appropriate selections. Finally, the user may browse the filtered results via the map and further probe the distribution of emerging variants or clusters.

#### Use Case 3: Spatial Distribution of Variant Prevalence and RR

**Figure 4:**
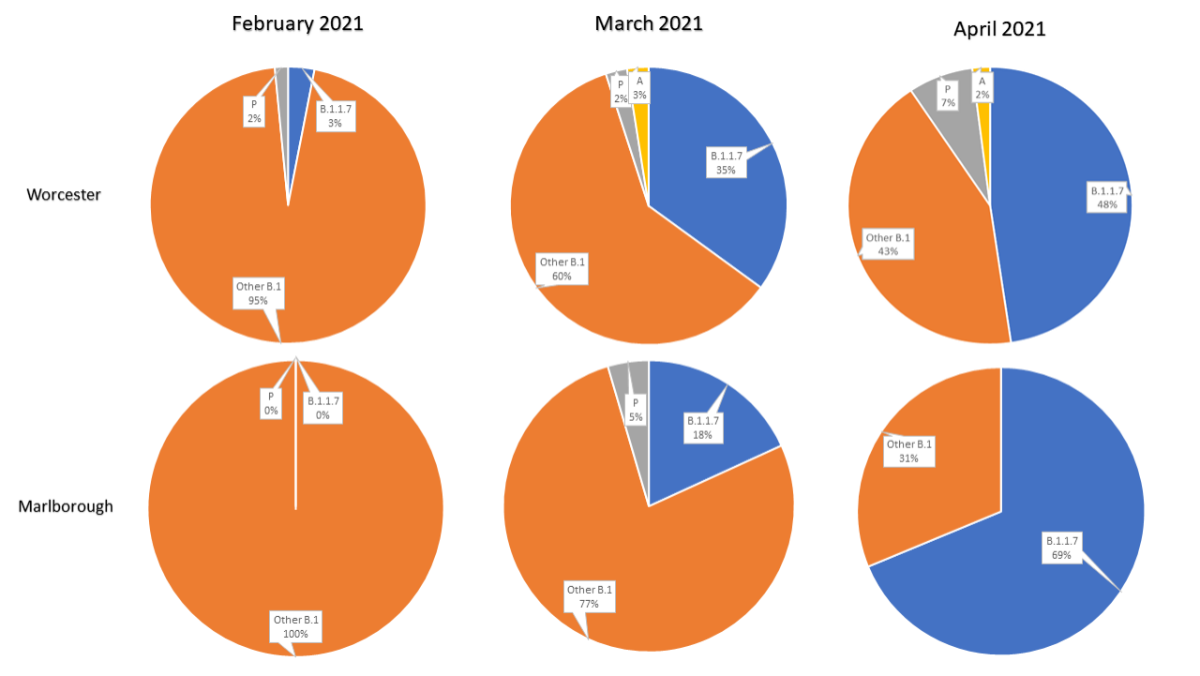
Variant tracking from February to April based on Massachusetts’ Graphical user interface for Geographic Information (MAGGI) data.

The objective of this use case was to measure the prevalence of variants. Users may use the strain lineage pie chart by configuring the zip code and time interval of interests. They can then observe variant transition dynamics across space and time by opting for a comparison to visualize the progression pattern categorized by demographics and vaccination status. For example, as shown in Figure 4, the Alpha variant progressed from 3% to 48% and from 0% to 69% in Worcester and Marlborough, respectively, between February and April 2021. A comparative visualization as represented in Figure 4 is not available on MAGGI’s user interface. To avoid distraction from an overly complicated pie chart with too many variants, which would be available in MAGGI, we created Figure 4, which only displayed the Alpha variant versus others.

We also calculated the RR of variants for selected geographical regions over time. The RR estimates provide insights on how prevalent a variant is in a specific location compared to the other areas. We used the risk from Central Massachusetts to determine our reference group, and then calculated the RR by dividing the risk from Worcester by the risk from the reference group. For example, we compared the RR of the Delta variant in Worcester with those in other Central Massachusetts regions between May and August 2021 (Table 1). We found that people living in Worcester, the largest city in Central Massachusetts, had a 214% increased risk of Delta infection in May 2021 than those living in other regions in Central Massachusetts. The risk of Delta infection increased rapidly through June and July, and individuals in both Worcester and other Central Massachusetts regions were 20 times more likely to contract the Delta variant in July compared to May 2021. We aimed to probe the social determinants of high RR in the early Delta era at the zip code level, but the small sample size after grouping by zip code and month limited the RR calculation at the zip code level.

**Table 1:** Relative risk of infection with the Delta variant from May to August 2021.

Month	Central Massachusetts except Worcester, RR^a^ (95% CI)	Worcester, RR (95% CI)
May	Ref^b^	3.14 (2.63-3.64)
June	5.76 (5.13-6.38)	12.75 (11.99-13.51)
July	20.29 (19.86-20.72)	22.56 (21.77-23.35)
August	22.33 (21.90-22.77)	22.34 (21.82-22.87)

^a^RR: relative risk.

^b^Ref: reference group.

## Discussion

### Principal Findings

We developed MAGGI, a secure platform that enables the spatiotemporal surveillance of the dissemination of SARS-CoV-2 variants. The UMass RDW and ArcGIS Ecosystem provided the foundational tool set for developing this platform. MAGGI is a research tool that is proving to be helpful for research into the socioeconomic and environmental determinants of the COVID-19 pandemic in our local region. Compared to other business intelligence tools, such as Tableau [45], Qlik [46], or Microsoft Power BI [47], the ArcGIS Ecosystem provides more powerful spatial capabilities, such as interactive spatial selection and hot spot cluster analysis. However, the interactive spatiotemporal solution we used can alternatively be developed through a traditional web-mapping approach using JavaScript and open-source spatial databases, such as PostGIS. However, that approach would require several months for a full stack developer and a geospatial team to create and deploy with limited change flexibility due to its resource-intensive coding nature. Although traditional web mapping provides better flexibility in user experience/user interface design, the cost to set up, administer, and support compared to the ArcGIS Ecosystem is greater. Furthermore, we presented our user interface designs in 3 separate meetings to epidemiologists and members of public health agencies, allowing them to provide feedback on the user interface. In the end, they were satisfied with the resulting user interface. For these reasons, we opted to take the ArcGIS route. With the recent development of ArcGIS Online Dashboard, the complexity to develop and deploy an interactive spatial dashboard for disease surveillance or public health management has been significantly reduced. The agility of the whole dashboard creation process enables epidemiologists to promptly determine where and when an infection outbreak occurs and which population it impacts the most.

Our development has multiple strengths. We accrued and sequenced over 5000 clinical samples and engaged genomic epidemiologists to generate questions examining variant transition dynamics in the context of social determinants and vaccination. Currently, the data sets used by the application are updated monthly. Our focus on providing high-resolution GIS is unique and relevant to current times given the COVID-19 pandemic. The Massachusetts DPH is interested in adopting MAGGI. With the continual emergence of fast-spreading SARS-CoV-2 variants such as Delta and Omicron, GIS technology around COVID-19 will only become more important as this technology advances and the adoption of the technology increases.

### Limitations

MAGGI is limited to UMMHC data. Therefore, we only captured patients who got their COVID-19 tests at UMMHC, and MAGGI is more likely to include patients who live near UMass Memorial Hospitals in Central Massachusetts. COVID-19 testing data becomes less accurate as we get further away from the hospital, thus limiting the generalizability of the findings using our tool. We hope to solve this issue by accessing data at the state level. In that case, sequencing data from all hospitals in Massachusetts will populate MAGGI, allowing researchers to make more informed investigations on variants, leading to breaking down the barrier. Another limitation of our tool is the small cohort size that it tracks, with 5000 specimens sequenced at this time.

### Future Direction

In the future, we plan to enhance the application in several ways. First, we will be automating data transfer and geoprocessing from the UMass Center for Microbiome Research into the MAGGI system to provide near real-time data analysis with a daily turnaround time instead of monthly. Second, we plan to aggregate street-level data to census geographical units (block groups, tracts) or zip code for the entire MAGGI system and create a public version of this tool so that it can be accessed by the general public. Third, we plan to create ArcGIS StoryMaps, a tool that enables researchers to create interactive narratives around their ideas with a strong sense of place [48]. We identified the ease and efficiency of making an informative presentation using StoryMaps when presenting to Massachusetts DPH officials. With collective feedback from epidemiologists and public officers, we will further polish and enhance the user experience of our tool. Finally, we will add new layers that could expose spatial clusters and hot spots of variants to enrich the geospatial mining ability to detect abnormal regions. Our goal is to enable deep phenotyping analytics using MAGGI to ultimately help our researchers identify actionable interventions.

## Abbreviations

ESRI: Environmental Systems Research Institute
GIS: Geographic Information System
MAGGI: Massachusetts’ Graphical user interface for Geographic Information
RDW: Research Data Warehouse
RR: Relative Risk
UMass: University of Massachusetts
UMMHC: UMass Memorial Health Center
ZCTA: ZIP Code Tabulation Areas
